# Long-Term Ultrasound Follow-Up of Thyroid Colloid Cysts

**DOI:** 10.1155/2014/350971

**Published:** 2014-04-22

**Authors:** Dong Wook Kim

**Affiliations:** Department of Radiology, Busan Paik Hospital, Inje University College of Medicine, Gaegeum-Dong, Busan 614-734, Republic of Korea

## Abstract

*Objective*. This study aimed to assess the interval changes of thyroid colloid cysts (TCCs) by performing long-term ultrasound (US) follow-up examinations. *Methods*. From 2007 to 2008, 437 patients underwent a lobectomy for the treatment of papillary thyroid microcarcinoma. Among them, 268 patients underwent 4 or more postoperative US follow-ups after surgery. This study investigated the prevalence and interval changes of TCCs ≥3 mm by using US follow-ups. *Results*. Among 268 patients, 35 (13.1%) had TCCs ≥3 mm by a preoperative thyroid US, and 6 (2.2%) had newly detected TCCs at a US follow-up. Through long-term US follow-up, the interval changes for TCCs were classified as follows: no interval change (*n* = 8), gradual increase (*n* = 8), gradual decrease (*n* = 5), positive fluctuation (*n* = 3), negative fluctuation (*n* = 6), disappearance (*n* = 5), and new detection (*n* = 6). None of the TCC cases had a TCC that was ≥10 mm at its largest diameter, and no patient complained of any relevant symptoms pertaining to the TCCs. *Conclusions*. In this study, TCCs demonstrated various interval changes, but no abrupt increase was found or acute onset of symptoms occurred.

## 1. Introduction

A thyroid colloid cyst (TCC) is a nonneoplastic thyroid nodule with a histology showing marked follicular dilatation and epithelial flattening [1]. A TCC contains a dense viscous material comprised of a concentrated solution of thyroglobulin; the cause of a TCC may be related to a defect in intraluminal thyroglobulin reabsorption [1]. A TCC is commonly detected by an ultrasound (US) examination of the neck or thyroid; however, its clinical significance is low because a TCC is not associated with a thyroid malignancy and is easily diagnosed by US examination because of its typical sonographic features [2, 3]. Many researchers and guidelines do not recommend US-guided fine-needle aspiration for TCCs because TCCs have inadequate cytology and typical sonographic features [2–8]. However, TCCs can result in an acute onset of symptoms, such as a palpable mass or pain, if they become larger than 2 cm at their largest diameter and if they develop an intralesional hemorrhage [2, 9]. Nevertheless, no data exists regarding the long-term interval changes of TCCs.

To the best of my knowledge, no previous study has employed a sonographic follow-up for TCCs by using a long-term follow-up interval of over 5 years. This study assessed the prevalence and interval changes of TCCs ≥ 3 mm at their largest diameter in postlobectomy patients who received treatment for papillary thyroid microcarcinoma (PTMC).

## 2. Materials and Methods

### 2.1. Patients

From January 2007 to December 2008, 437 patients (374 women and 63 men; range 18–72 years; mean age 45.5 years) underwent lobectomy for the treatment of papillary thyroid microcarcinoma (PTMC) in our hospital. All the patients underwent thyroxine-replacement therapy after lobectomy. Among them, 268 patients (227 women and 41 men; range 18–70 years; mean age 45.5 years) were included in this study that had 4 or more postoperative follow-up US examinations after lobectomy. The institutional review board approved this study (IRB 13-158), and informed consent was waived for this retrospective study.

### 2.2. Thyroid Ultrasound

An experienced radiologist performed the thyroid US examinations using a high-resolution ultrasound instrument (HDI 5000 or iU 22; Philips Medical Systems, Bothell, WA, USA) equipped with a 5–15 MHz linear probe. A TCC was defined as a pure thyroid cyst with intracystic comet-tail artifact(s). To clarify the sonographic detection and to facilitate the evaluation of an interval change for the TCCs, only TCCs ≥ 3 mm at their largest diameter were included in this study. In the case of multiple TCCs, only the dominant TCC (i.e., the largest TCC) was included in this study.

In our hospital, the routine US follow-ups of postlobectomy patients who received treatment for PTMC were performed as follows: the initial 3 US follow-ups were performed at a 1-year interval, and the later US follow-ups were performed at 1- or 2-year interval depending on patient agreement. In this study, patients were excluded if they had 3 or less US follow-ups or did not have at least 1 US follow-up after the 5-year or more interval from thyroid surgery.

### 2.3. Sonographic Classification

An interval increase or decrease for each TCC was defined as a positive or negative interval change of 10% or more in the largest diameter of a TCC during the US follow-ups. On the basis of the US follow-ups, each TCC was retrospectively classified into 1 of 7 diagnostic categories by a single radiologist: (1) no interval change: an interval change of <10% occurred in the largest diameter of the TCC; (2) gradual increase: an interval increase occurred in the TCC at its largest diameter but did not have any fluctuations during the sequential US follow-ups; (3) gradual decrease: an interval decrease occurred in the TCC at its largest diameter but did not have any fluctuations during the sequential US follow-ups; (4) positive fluctuation: an interval decrease occurred in the TCC during the early US follow-ups and an interval increase occurred in the TCC during the late US follow-ups; (5) negative fluctuation: an interval increase occurred in the TCC during the early US follow-ups and an interval decrease occurred in the TCC during the late US follow-ups; (6) disappearance: no TCC was visualized during the US follow-ups; and (7) new detection: a new TCC was observed during a late or the last US follow-up.

## 3. Results

**Figure 1:**
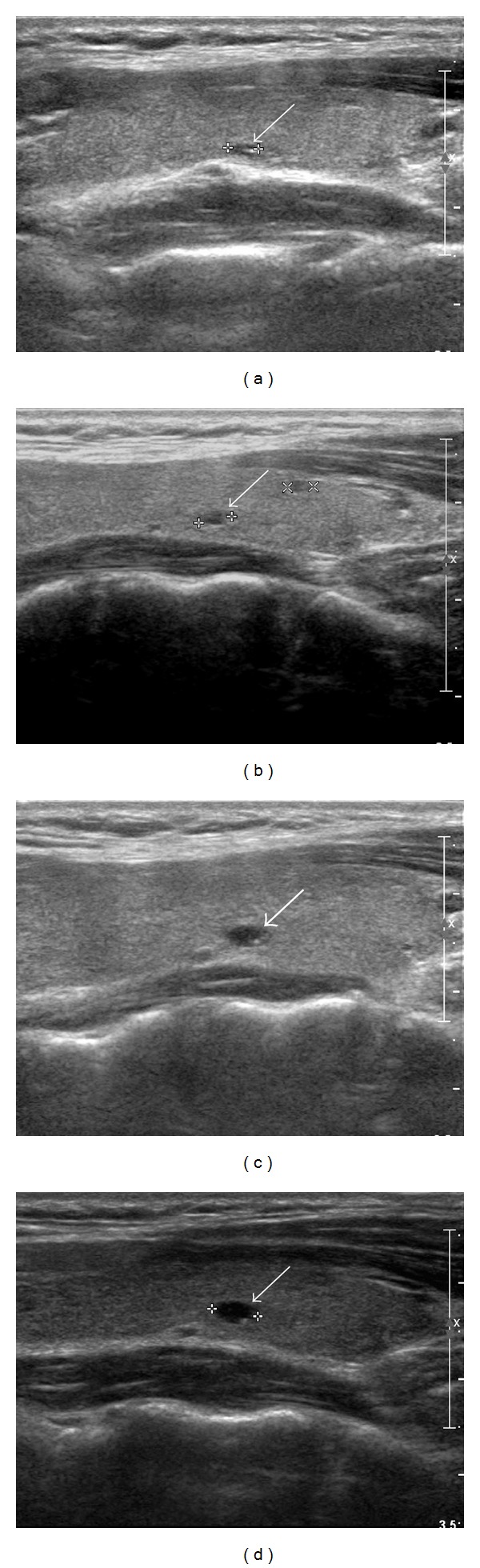
A 27-year-old woman with gradual increase in a thyroid colloid cyst. Preoperative longitudinal sonogram (a) shows a colloid cyst with an anechoic nodule and a comet-tail artifact in the left thyroid lobe (arrow, 3.2 mm at its largest diameter). Upon sequential US follow-ups, this colloid cyst demonstrates a gradual increase at a 1-year (b) (arrow, 3.5 mm at its largest diameter), a 3-year (c) (arrow, 4.7 mm at its largest diameter), and a 5-year (d) (arrow, 5.1 mm at its largest diameter) postlobectomy.

Of the 437 patients, 268 patients (61.3%) underwent a long-term follow-up with US examinations (range 60–78 months; mean 65.7 months). Of the 268 patients, 41 patients (15.3%; 32 women and 9 men; range 24–66 years; mean age 42.2 years) had TCCs ≥ 3 mm at the largest diameter of the TCCs (range 3.0–8.2 mm; mean 3.9 mm). Among them, 35 patients (13.1%) had TCCs during both the preoperative thyroid US examination and US follow-ups; however, 6 patients (2.2%) had TCCs only during the US follow-ups. Through the preoperative thyroid US examinations and US follow-ups, the interval change for the TCCs was classified as follows: no interval change (*n* = 8), gradual increase (*n* = 8) (Figure 1), gradual decrease (*n* = 5), positive fluctuation (*n* = 3), negative fluctuation (*n* = 6), disappearance (*n* = 5), and new detection (*n* = 6). The sonographic results for a total of 41 TCCs are summarized in Table 1.

Of the 41 patients with TCCs, no patient had a TCC ≥ 10 mm at its largest diameter during both the preoperative thyroid US examinations and US follow-ups. During the thyroid US follow-ups, no TCCs demonstrated an increase that was 2 or more times of their largest diameter. Further, no patients had any sonographically suspected intracystic hemorrhages or complained of any relevant symptoms.

## 4. Discussion

The clinical significance of a TCC is very low because a TCC is not associated with a thyroid malignancy and is easily diagnosed by a thyroid US examination [2–4, 7]. In particular, a TCC can be sonographically diagnosed because a TCC has some typical sonographic features (i.e., pure thyroid cyst with intracystic comet-tail artifacts) [2], and thus many radiologists do not recommend US-guided fine-needle aspiration for a TCC [2–4, 7]. Sometimes, a TCC results in an intracystic hemorrhage, and thus patients can complain of local pain or a palpable neck mass [2]. However, a long-term interval change in a TCC has not been reported. This study attempted to investigate the long-term sonographic interval changes of TCCs in postlobectomy patients who were treated for PTMC.

In the present study, TCCs had various interval changes, although no TCC had largest diameter ≥ 10 mm. Among them, the most common patterns were no interval change (19.5%) and gradual increase (19.5%). However, the incidence of a gradual decrease (12.2%) and disappearance (12.2%) was not low. In this study, various interval changes were observed with respect to TCCs, but none of the TCCs demonstrated an abrupt increase or acute onset of symptoms. Therefore, the author believes long-term US follow-up for TCC is unnecessary.

High-resolution thyroid US examinations have been used worldwide for the evaluation of thyroid nodules that resulted in the general acceptance of certain characteristics that mark benign and malignant thyroid nodules [4–8]. A thyroid US examination has been considered the most accurate imaging tool for evaluating thyroid nodules despite its operator dependency [4, 7, 8]. In this study, an experienced radiologist who had 10 years of experience in thyroid US (>2500 cases/year) performed all US examinations. Only a thyroid nodule showing an anechoic thyroid nodule with comet-tail artifact was considered to be a TCC.

There were several limitations in this study. First, of the 437 patients, 169 patients (38.7%) were not included because of insufficient US follow-ups. Further, only patients who underwent lobectomy because of PTMC were included; thus, a selection bias was possible. In addition, a comparison analysis was not performed for TCC interval changes secondary to thyroxine-replacement therapy during the maintenance periods because of the small number of cases in each category. Several reports have shown that shrinkage of thyroid nodules occurs more frequently in patients with long-term thyroid-stimulating hormone suppression than in untreated patients [10, 11]. For clarity, a large-scale study involving a general population may be required. Finally, a single radiologist performed all the thyroid US examinations, including preoperative thyroid US and postoperative US follow-ups.

In conclusion, TCCs show various interval changes in the patients who underwent lobectomy for papillary thyroid microcarcinoma, but TCCs rarely have an abrupt increase or acute onset of symptoms.

## Figures and Tables

**Table 1:** The sonographic results of 41 patients with thyroid colloid cysts.

Interval change	Location	Mean size (mm)
No interval change (8)	Rt (4), Lt (4)	4.6
Gradual increase (8)	Rt (3), Lt (5)	3.4
Gradual decrease (5)	Rt (3), Lt (2)	4.3
Positive fluctuation (3)	Rt (0), Lt (3)	3.7
Negative fluctuation (6)	Rt (1), Lt (5)	4.8
Disappearance (5)	Rt (1), Lt (4)	3.2
New detection (6)	Rt (3), Lt (3)	3.8

*Note.* Data presented in parentheses are number of each item. Rt: right; Lt: left.
